# The use of a surgical boot camp combining anatomical education and surgical simulation for internship preparedness among senior medical students

**DOI:** 10.1186/s12909-022-03536-y

**Published:** 2022-06-15

**Authors:** Jifeng Zhang, Prince Last Mudenda Zilundu, Wenbin Zhang, Guangyin Yu, Sumei Li, Lihua Zhou, Guoqing Guo

**Affiliations:** 1 Department of Anatomy, Basic Medical College, Jinan University, Guangzhou, China; 2 Department of Medical and Dental Sciences, College of Dentistry, Ajman University, Ajman, United Arab Emirates; 3 Department of Anatomy, Sun Yat-Sen School of Medicine, Sun Yat-Sen University, Shenzhen, China; 4 Department of Surgery, The First Clinical Medical College, Jinan University, Guangzhou, China

**Keywords:** Medical students, Surgical boot camp curriculum, The core competitiveness of clinical practice

## Abstract

**Background:**

Senior medical students feel unprepared for surgical procedures and care for surgery patients when they begin their internship. This study sought to introduce and evaluate a surgical boot camp training for senior medical students.

**Methods:**

A 44-h surgical boot camp program of lectures on clinical practice simulation, anatomical dissections, and simulated operation on cadavers was designed, implemented, and evaluated during the 2018 to 2019 academic year. A self-administered questionnaire was used to assess students’ perceptions of the content, delivery, and self-confidence. The mini-Clinical Evaluation Exercise (mini-CEX) and the Operative Performance Rating System were used to assess skills essential to good clinical care and to facilitate feedback.

**Results:**

Over 93% of the students were satisfied with the surgical boot camp, training equipment, and learning materials provided. After six sessions of training, 85.3% reported gaining self-confidence and performed better in some surgical procedures such as major gastrectomy. The mini-CEX scores suggested significant improvement in the students’ clinical skills, attitudes, and behaviors (*P* < 0.01). Ninety-eight percent of students felt that the anatomical knowledge taught met their needs. The scores of the Operative Performance Rating System suggested that the students’ surgical skills such as instruments handling, incising, treatment of surrounding tissues (blood vessels, nerves), and smoothness of the whole operation had increased significantly following the surgical boot camp (All *P* < 0.01).

**Conclusion:**

The surgical boot camp curriculum improved students’ satisfaction and confidence in core clinical practice competencies. Therefore, medical schools the world over should continue to seek ways to bridge the gaps between pre-clinical, clinical, and internship training.

**Supplementary Information:**

The online version contains supplementary material available at 10.1186/s12909-022-03536-y.

## Background

Chinese medical schools generally follow a traditional medical education format whereby the basic medical sciences curricula mainly deal with the general principles of disease occurrence while the clinical-stage curricula pay more attention to the clinical symptoms, diagnosis, and treatment of diseases [1]. This creates a big gap between the basic and clinical medicine curricula with no suitable plan to cover it. This scenario makes medical students find it difficult to appreciate how the basic medical sciences could assist them better understand the pathophysiology and treatment of diseases [2]. While attempts at integrated curricula have sought to bridge this gap [3–5], there are numerous reports outlining implementation challenges across the world [6–8]. Disjointed curricula also cascade into ill-prepared graduates entering internships [9, 10].

World over, medical schools are inundated with reports of dissatisfaction with the preclinical, clinical, and internship transitions from both students as well as instructors who are yearning for solutions [5, 11]. For instance, after entering the hospital for clerkship and internship, medical students have to spend a lot of time and effort trying to transform the preclinical theoretical knowledge into clinical applications. Some studies have reported senior medical students and interns forgetting much of preclinical subjects such as anatomy [12–14]. Yet they must translate the understanding of the spatial positions of anatomical structures to improving surgical skills, especially for operations and clinical examinations [15–18]. In 2013, the Chinese government launched the “Guidelines for Standardized Resident Training” that sought to guide training objectives, dictate rotation length requirements, rationalize training content, and act as reference material for healthcare institutions [19]. The guidelines became compulsory in 2020 and it becomes imperative to ensure that senior medical students have a seamless transition into internships. The medical training framework in China involves basic science (year 1–2) and clinical medicine (years 3–5 or 6) training followed by a year-long internship. During this internship, the final year (year 5 or 6) medical students rotate in medical specialties of the medical schools’ affiliated hospitals and then sit for a post internship exam before they can graduate from their respective universities. After graduation, the medical graduates then transition into government and other designated hospitals for a three-year long housemanship (internship/junior resident medical officers) where they work under supervision. This unique settings lead to three transitions namely, basic science/clinical medicine, clinical medicine/1-year internship and 1-year internship/3-year housemanship. The present study focused on the transition of senior medical students from clinical years into the one-year internship preparation. This period represents a big opportunity to bridge the gap between University learning and the hospital-based hands-on apprenticeship with patient access and bed-side management.

From the Halstedian model of surgical training to the goal-driven training model, surgical education has gone through more than 100 years [20]. The traditional Halstedian training model of "see one, do one, learn one" was a system in which medical school graduates entered a university-sponsored, hospital-based surgical training program that, over a several years of increasing responsibility which gradually resulted in the training of young surgeons well versed in anatomy, pathology, bacteriology, and physiology [21]. The end product of the training program were near-total independence and autonomous graduates first observed, then performed, and finally demonstrated specific surgical procedures. However, it is gradually being replaced by a new one, the model of "simulate one, do one, teach one" especially in cases involving invasive procedures and high-risk care [17, 22–25]. Lev S. Vygotsky’s sociocultural theory of cognitive development has been applied to medical education to support learning and surgical skill acquisition thus provide a framework for enhancing surgical education [26, 27]. Vygotsky’s concept of “zone of proximal development” refers to the space between what learners have mastered and what they should master in the next developmental stage [28]. This concept is applicable to medical students’ quest to master surgical skills competency required for safe clinical practice when transitioning from medical school into internships. Under this Vygotsky’s sociocultural theory, the skilled surgeons and anatomy teachers assess medical students’ skills deficits as well as facilitate the acquisition and hand-over of appropriate surgical skills. The present surgical boot camp curriculum, which included surgical simulation, was designed to teaching surgical skills in line with Vygotsky’s assisted performance concept and the following four stages of the zone of proximal development [28]. During stage one, performance is assisted by more capable others such as surgeons and help is dependent on the nature of the task and the competency level of the learner. For instance, the surgeons provide directions or modeling, and the medical student’s responses are often acquiescent or imitative. The second stage involves handing over of control or assistance from the surgical expert to the apprentice (medical student) and the performance of the task itself effectively handed over to the learner. Stage three is where performance is developed, automatized whilst task execution is smooth and integrated. At this stage, the learner has emerged from the zone of proximal development into the developmental stage for the task and assistance from the expert is no longer needed. Stage four is where de-automatization of task performance causes relapses back into the zone of proximal development. During lifelong learning, the cycles of regulated zone of proximal development may be repeated as the medical students turned professionals gain new capacities.

Fortunately, recent advances in web-based education, virtual reality, and high-fidelity patient simulation have come in handy to enhance medical and surgical education [25, 29] as well as the transition from medical school into internship or residency [30]. These advances, aimed at bridging the gaps between theoretical knowledge acquisition and clinical practice, have been integrated with reported successes in improving clinical skills in some medical curricula [30, 31]. In China, simulation and boot-camp type training in medical education has been lagging, but is getting adopted as part of modern curriculum reforms [32].

In 1999, the United States Accreditation Council for Graduate Medical Education established six core competencies to be achieved during residency, namely medical knowledge, patient care, interpersonal and communication skills, professionalism, practice-based learning and improvement, and systems-based practice [33]. As a result, questions have lingered on how to achieve the core goal of cultivating clinical competencies or how to transform theoretical medical knowledge into effective clinical practice to make students fit for the clinical work as soon as possible. Major curricular reforms have turned to employing horizontal and vertical curricular integration to bring together the basic and clinical science disciplines [2, 7]. Elsewhere, surgical boot camps are effective means to improve clinical practice, self-confidence, and surgical skills [34–36]. However, the implementation of pre-internship boot-camps bringing together anatomical dissections, simulation, and surgical practice has not been widely reported in China [37].

Most medical curricula are mainly geared towards fostering the students’ clinical practice abilities and operation skills through simulation to help them better adapt during internships. However, they rarely integrate simulated surgery with anatomical dissections, especially the fresh frozen cadavers. In the present study, a special surgical boot camp aimed at better integration of theoretical preclinical knowledge with clinical practice among senior medical students was designed, implemented, and evaluated. The curriculum presents a good model for medical students to transition into interns faster and more effectively.

## Methods

### Participants

The invitation to participate in the surgical boot camp is a flagship of the university that is offered to senior medical students with a total enrollment of 106 students. A total of 24 students in the training camp were 4^th^-year undergraduate medical students during the 2018/2019 academic year (22.6% participation rate). The participants covered dissection based gross anatomy (regional and systemic) alongside histology (with light microscopy practicals) and embryology in first and second years of their studies as described previously [38]. Participation in the surgical boot camp was offered as part of the overall internship curriculum for students who are in the third year or above over the past 4 years. Participation in this study was voluntary. Two clinicians and two anatomy teachers took part in the surgical boot camp program for the 2018 to 2019 academic year. The clinicians are general surgeons in the First Clinical College of Jinan University with more than 10 years of work experience. The anatomy teachers are domiciled in the Department of Anatomy of Medical School of Jinan University with more than 5 years of teaching experience.

### Surgical boot camp design

As a transition tool from medical school into clinical practice, the present surgical boot camp curriculum aimed to prepare medical students for the 1-year pre-graduation internship. The teaching plan for this curriculum was reviewed and approved by the Academic Affairs Office of the Medical School of Jinan University.

Unlike a typical bootcamp that takes place in one seamless period, the present modified bootcamp ran alongside the normal teaching and part of the off-semester break covering two months. The curriculum was composed of didactic, simulation, and practical sessions covering topics in four major categories: lectures for acute patient management, clinical practice simulation, anatomical dissections, and mock surgical operations using fresh frozen cadavers. The use of fresh frozen cadavers has been described in a review by Song and Jo [39]. This curriculum was drawn from the surgeons’ longstanding involvement in anatomy teaching at our school as described previously [38]. The model used in the present study comprised:

(i). Clinical sciences lectures: These were taught by clinicians and the topics included pre-and post-operative management, progress in diagnosis, and treatment. Pre-operative and post-operative management mainly included topics such as preparation for surgical operation, tissue examination, pain, infection, incision care, nutrition, and complications management. The progression of diagnosis and treatment included topics such as the etiology of the disease, clinical manifestations, diagnosis, and differential diagnosis, surgical methods.
(ii). Clinical practice simulation: focused on the patient’s history, the physical examination, the laboratory examination, the treatment planning, and communication with patients/significant others. Students are supposed to finish the entire process such as physical examination, laboratory examination, and other aspects of clinical practice at the University’s clinical simulation center.
(iii). Anatomical dissections: These were available in two parts. The first part was the applied clinical anatomy taught by the anatomy teacher. It was mainly concerned about the location and relations of the anatomical structures as well as their clinical applications. The other part involves the actual dissection of cadavers such as separation and identification of the viscera and related structures in the region of interest.
(iv). The surgical operation sessions: Use of fresh frozen cadavers [39] also consisted of two parts. The first part involved the training of surgical skills such as basic skills like suturing, knotting, tube placement, and drainage. The second part is the operation simulation also on fresh frozen cadavers according to standard surgical procedures. These operations were performed in the University’s surgical simulation center. The types of surgery chosen for this surgical boot camp included appendectomy, cholecystectomy, splenectomy, intestinal anastomosis, inguinal hernia repair, and subtotal gastrectomy. The students were encouraged to use their free time to practice stitching or knotting and their mastery was evaluated before the operation simulations.

The surgical simulation session was held during the last 2 weeks of the academic year, just before the beginning of clinical duties. The clinical sciences lectures, clinical practice, and anatomical dissection sessions were held during the protected time designated exclusively for the modified surgical boot camp throughout one academic semester. These were typically 1–4-h sessions per week spread over for 2 months. The total time of the curriculum was 44 h (Additional file 1: Appendix A).

### The survey

The 3 major skill categories addressed during the surgical boot camp were used as a framework to develop the survey questionnaire along with a review of relevant literature [40–43]. Survey questions were developed based on previous literature and reviewed by a panel of education experts, including the surgical boot camp program leadership. The survey on each session adopted a 5-point Likert scale (Additional file 1: Appendix B). The survey questionnaire was composed of 18 items: 3 questions were about the curriculum preparation, and 15 questions covered the curriculum content, delivery, and evaluation. The questionnaire was distributed to the students at the end of the boot camp, in which they were asked to evaluate curriculum topics and report on their overall level of satisfaction with the surgical boot camp using a Likert-type scale (strongly disagree, disagree, neither disagree nor agree, agree, or strongly agree). All 24 students provided their written informed consent to participate in the study before they filled out the questionnaire.

The clinical practice ability was assessed using the Mini-Clinical Evaluation Exercise (CEX) scale which assessed 6 aspects, namely, the medical interviewing skills, physical examination skills, humanistic qualities/professionalism, clinical judgment, counseling skills, and organization/efficiency (Additional file 1: Appendix C) [44, 45]. The assessments were carried out after the first and 6^th^ (last) training sessions. Surgical skills were assessed using the Operative Performance Rating System (OPRS) which collects data on 8 aspects namely incision, exposure, structure confirmation, reparation, instrument handling, respect for tissue, time and motion, as well as operation flow (Additional file 2: Appendix D) [46, 47]. The overall performance of each operation was assessed on-site by two clinicians at the end of the first and 6^th^ (last) training sessions. All survey instruments were reviewed and approved by the Medical Ethics Committee of Jinan University.

### Statistical analysis

The measurement data were expressed as mean ± standard deviation. The statistical analysis was performed using the SPSS 26.0 software (IBM Inc. USA). A *P* < 0.05 was considered statistically significant. Repeated-measures ANOVA was used to assess differences in the total 6 operation scores, and comparisons between different time points were tested by paired *t*-test. The unpaired *t*-test was used to compare the clinical practice ability before and after training.

## Results

### Responses to surgical boot camp

**Fig. 1:**
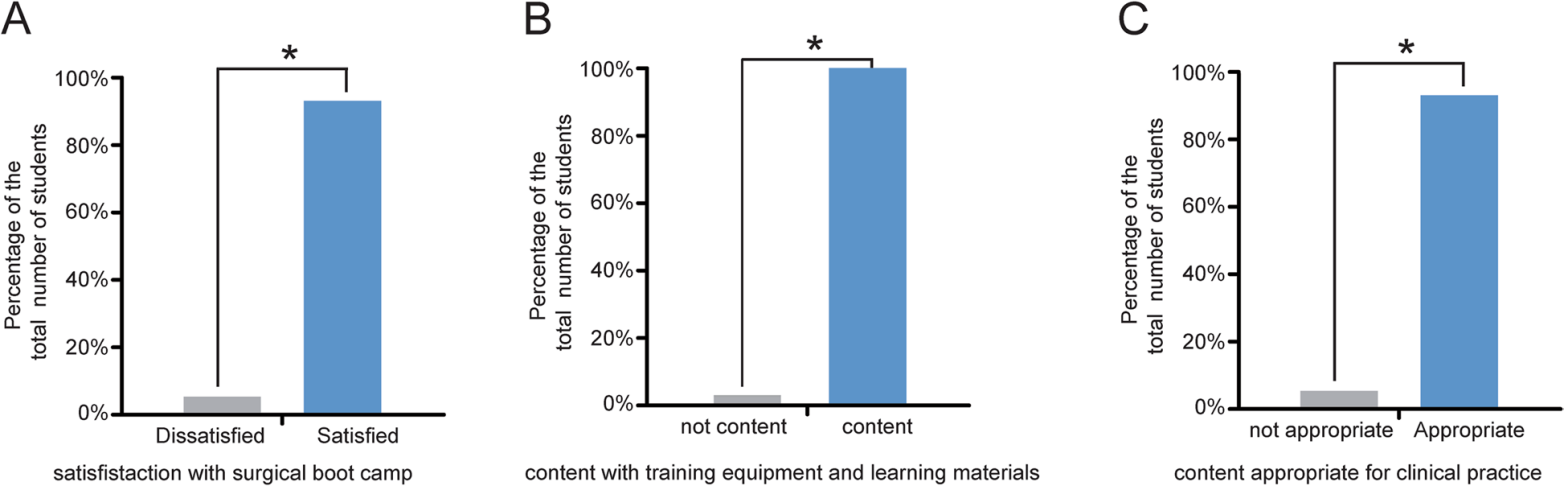
Students’ overall satisfaction with the surgical boot camp curriculum components. **A** The majority of the students were satisfied with the overall surgical boot camp training. *n* = 24. **B** Level of contentment with the training resources provided. **C** Appropriateness of content delivered during the surgical bootcamp

All students responded to the questionnaire survey. They were 12 males and 12 females with an average age of 23.5 ± 0.9 years. The Cronbach’s alpha for the survey was 0.87 demonstrating that the questionnaire was reliable. The survey results showed that more than 93% of the students were satisfied with the surgical boot camp (Fig. 1A). Among 24 students, 95.7% of them were content with the training equipment and learning materials provided during the boot camp (Fig. 1B). For the content set, 92.2% of students felt it was appropriate for them, and that the training curriculum gave them a valuable clinical practice experience (Fig. 1C).

**Fig. 2:**
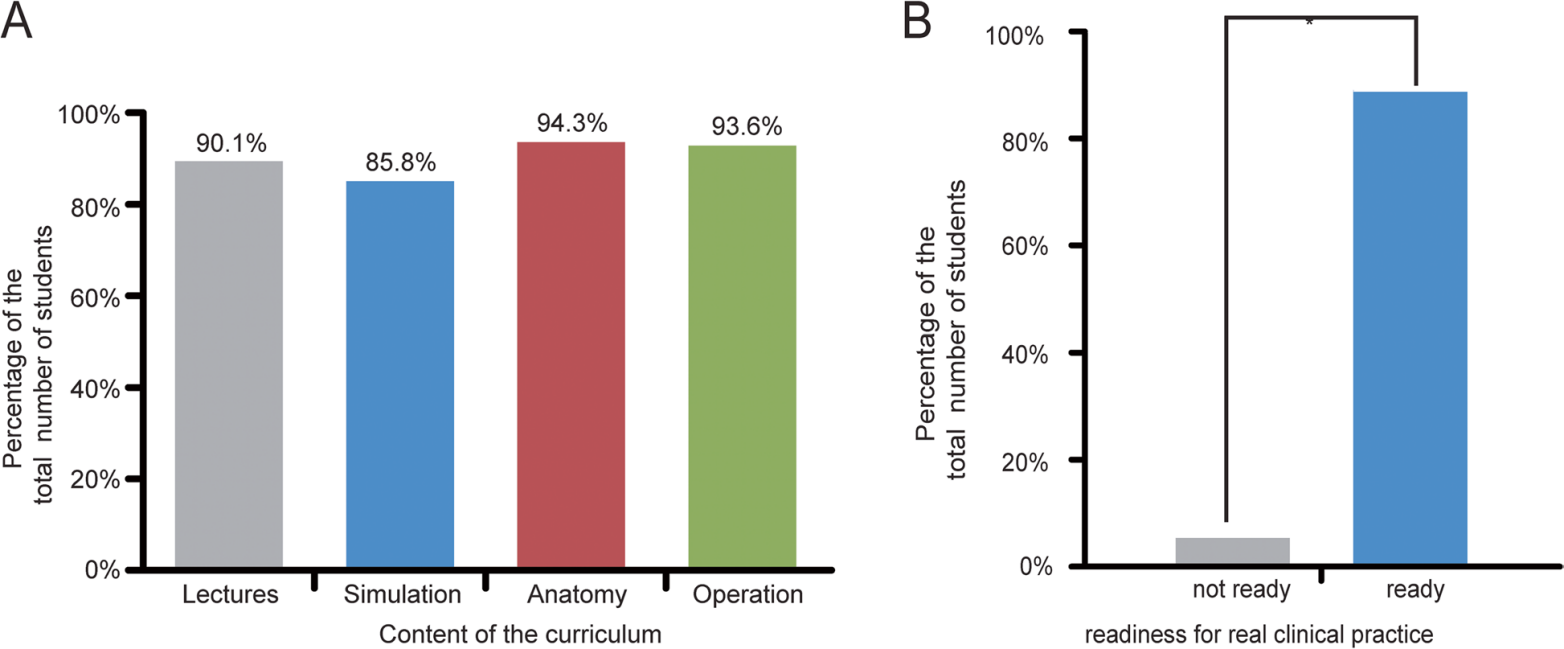
Students’ preferences for the surgical boot camp components and readiness for clinical practice. **A** Over 85% of the students felt positive about the lectures, simulations, anatomical dissections, and operations content. **B** Most of the students felt ready to begin internship after attending the bootcamp

As shown in Fig. 2A, most of the students (90.1%) felt that clinical sciences lectures covered the main aspects of clinical practice. The majority (85.8%) of students also felt that surgical simulation practice was rich enough to meet their curriculum objective needs (Fig. 2A). In addition, Fig. 2A shows that 94.3% of students were satisfied with the anatomical dissection content whilst the remaining minority felt the dissection content was inadequate. Furthermore, 93.6% were satisfied with the operation simulations compared to a few that were not (6.7%) (Fig. 2). Overall, over 85% of the students felt ready to enter the wards for real clinical practice (Fig. 2B).

### Improved ability of clinical practice

**Fig. 3:**
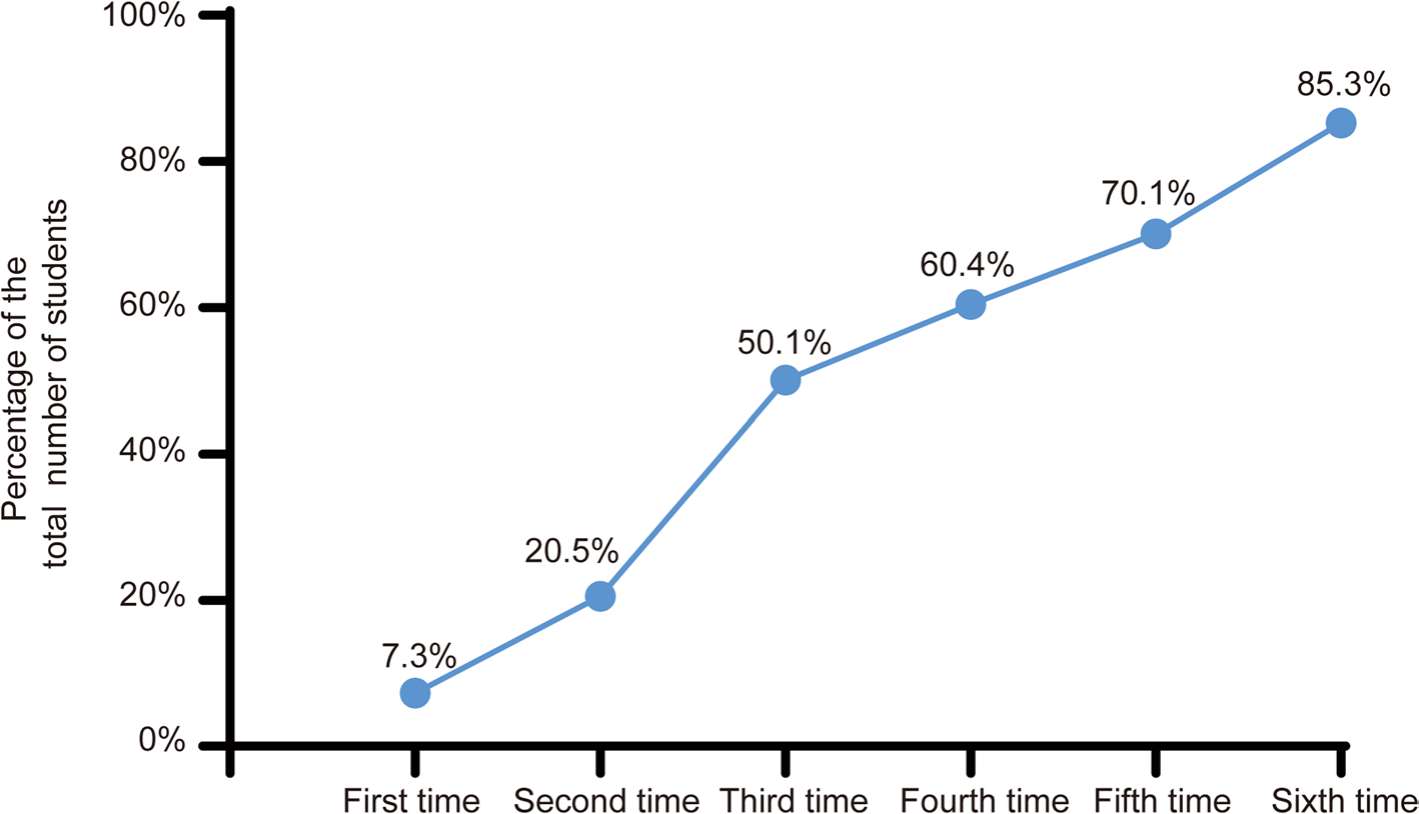
Teacher’s evaluation of the performance of students during the surgical bootcamp sessions.There was a significant increase in the number of students possessed better procedural skills and were self-confident compared with the first-session evaluation time

To clarify students’ clinical practice ability, the teachers’ evaluation of the student’s performance after the first up to the sixth training sessions were compared. In the first evaluation, the teaching faculty observed that only about 7.3% of students demonstrated better clinical practice skills while 93.5% of students were deemed inadequately skilled. However, significant improvements were recorded up to the sixth session of training, with the teachers observing that 85.3% of students possessed better procedural skills and were self-confident compared with the first-session time (*P* < 0.05). After the boot camp training, the teachers were unsatisfied with the performance of under 15% of students (Fig. 3).

**Fig. 4:**
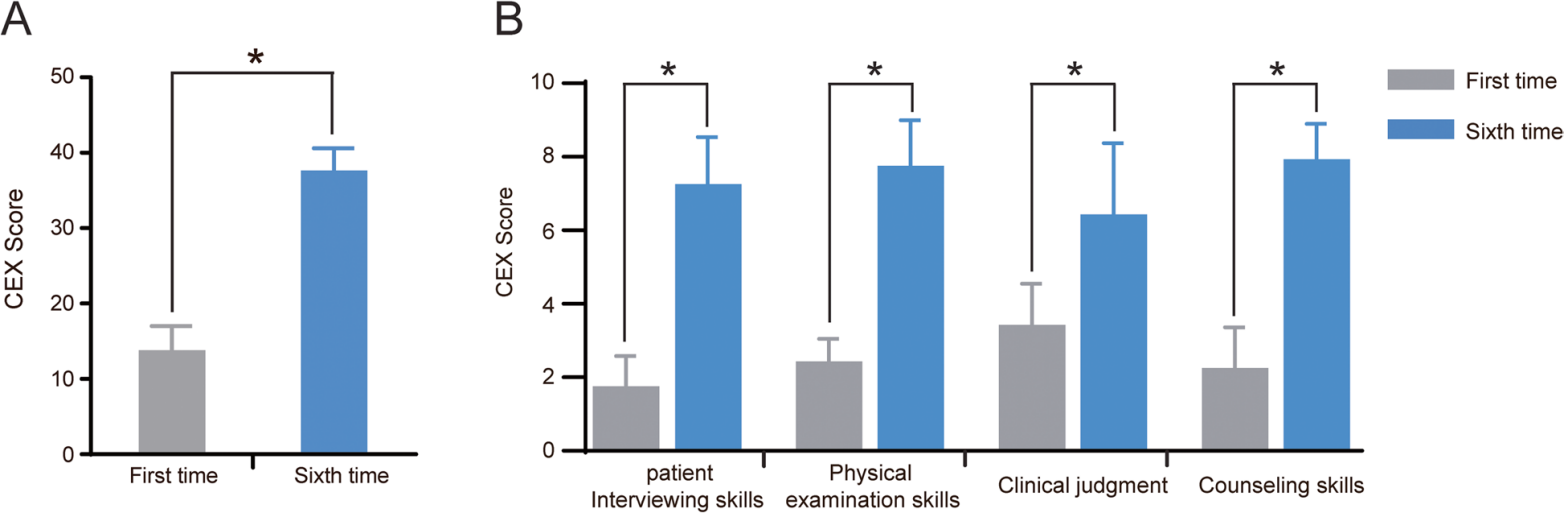
The score of overall clinical competence in students evaluated by mini-CEX. **A** Significant improvement of overall competency after 6 weeks of training. **B** Changes in patient interviewing skills, physical examination skills, clinical judgement, and counseling skills (first versus sixth time abilities)

Next, we evaluated the overall clinical competence using the mini-CEX. As depicted in Fig. 4A, the students’ overall clinical practice abilities had significantly improved following the successive training sessions (*P* < 0.05). Furthermore, we analyzed the scores of the interviewing skills, physical examination, clinical judgment, and counseling skills. The results indicated that the scores of these four skills had significantly improved at the end of the boot camp when compared with those obtained after the first training session. Specifically, the scores of physical examination and counseling skills had reached excellent levels (Fig. 4B).

### Improved operation skills

**Fig. 5:**
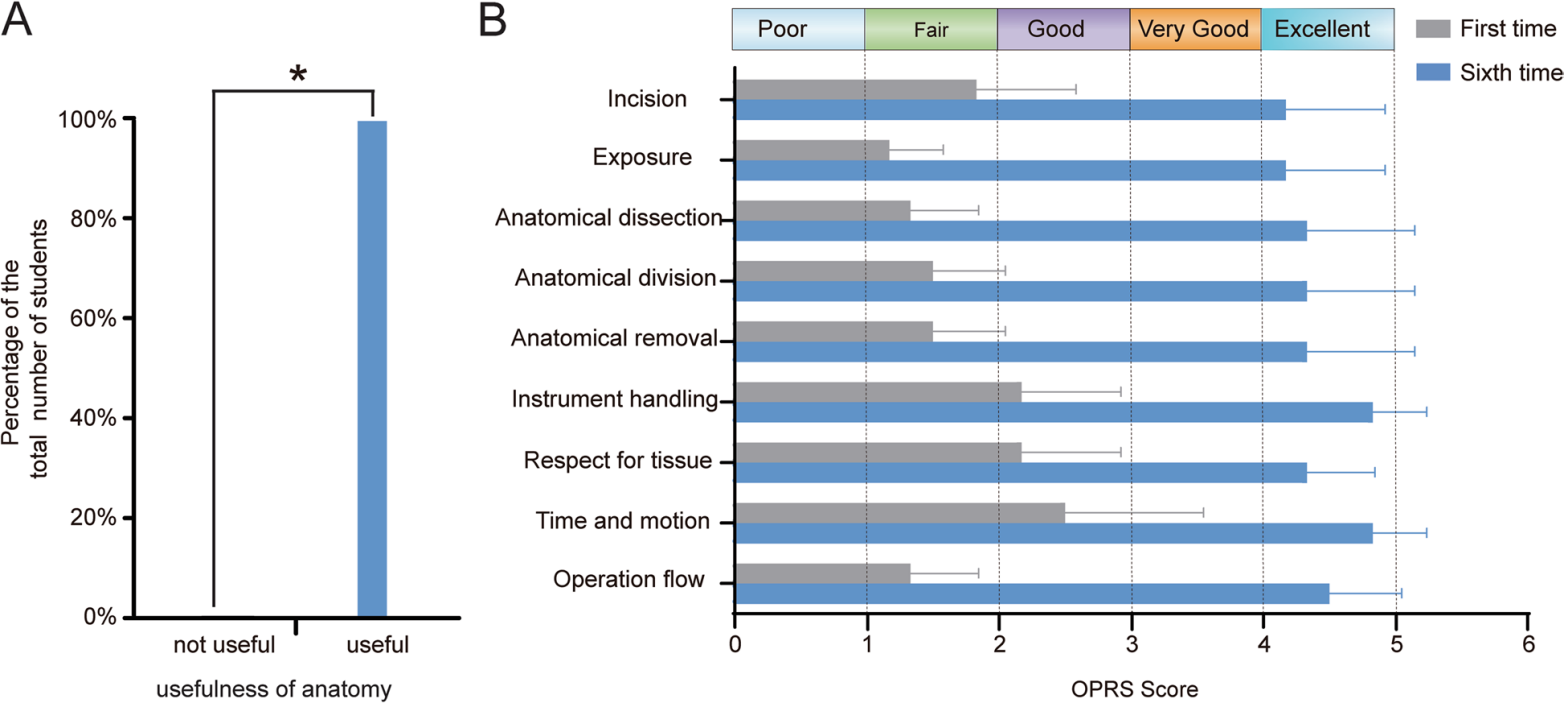
The benefit of anatomical knowledge towards simulation operation and the Operative Performance Rating System (OPRS). **B** Overall improvement OPRS scores in eight of nine operative procedures: incision, exposure, structure confirmation, reparation, instrument handling, respect for tissue, time-motion, as well as operative flow

To determine whether students’ surgical skills had improved after anatomical and simulated surgery training, we first investigated the students’ view of the anatomy knowledge post-training. The present results revealed that 98% of students reported that the anatomical knowledge gained during the boot camp training sessions was useful and would meet their simulated surgery needs (Fig. 5A).

Next, we evaluated their surgical operation skills from the surgical incision to the end of the operation using the OPRS. Three-quarters of the students hesitated to make an incision during the first training session, but gradually became accurate as well as decisive, and the exposure of the structures became clearer and more accurate. Moreover, because of proficiency in using the surgical instruments and the treatment of tissues, muscle, and blood vessel separation gradually became more accurate, whilst the ineffective movements gradually diminished. At the end of the training sessions, the entire surgical process became smooth and efficient. Judging from the OPRS overall performance scores, the curriculum had statistically significant beneficial effects on the students’ perception of their abilities. The scores of OPRS had increased significantly from the first to sixth operation. Although the major gastrectomy, the last operation, is a relatively difficult operation, the students still performed well (Fig. 5B).

## Discussion

In the present study, a surgical boot camp for senior medical students encompassing lectures on acute care clinical management, anatomical dissections, and simulated surgery was designed, implemented, and evaluated. The curriculum aimed to improve students’ clinical knowledge and surgical practice ability. Following training, the students’ clinical practice and operation skills had generally improved. This study demonstrated that comprehensive training of senior medical students helped them improve their satisfaction, confidence as well as clinical, technical, and surgical/procedural skills.

The results of this study showed that nearly all the students were satisfied with the surgical boot camp curriculum. This suggested that students hugely benefited from the implementation of the program, hence the positive feedback. The surgical boot camp was aimed at preparing senior medical students to smoothly transition into clinical practice as interns. Before entering the hospital as interns, the medical students often face challenges on how to apply the medical knowledge learned in collegiate courses to clinical practice [48, 49]. They have to deal with how to communicate with patients to obtain valuable history or to diagnose what disease the patient has according to clinical manifestations. They also must formulate the treatment plan and even figure out how to support the patients if they need surgery. Most students often fail to integrate anatomy knowledge into an operation [48, 50–52]. The present boot camp curriculum sought to bridge this gap by teaching students how to integrate scattered medical knowledge to solve a specific problem. Therefore, this aspect of the present training could be the reason medical students were satisfied with the content of the surgical boot camp. The present findings of positive evaluation have been echoes in several previous studies [53, 54]. However, the minority who did not positively evaluate the boot camp sessions are probably not interested in surgery or may have felt competent without such a training. Another explanation according to the Vygotskyan model used to design this boot camp curriculum could be that these students did not have a huge gap between what they had already mastered (comfort zone) and what they needed to master (zone of proximal development frontier) [55, 56]. A qualitative research design would illuminate the medical students’ lived experiences and more in-depth understanding of this phenomenon. Furthermore, future studies should find ways of mainstreaming vertical integration of the curricula while designing boot-camp courses tailored to the graduating students’ residency matching or intended specialization as well as focus on pretest/posttest research designs including use of control groups as recommended earlier [31].

One of the key aspects of the surgical boot camp is to improve students’ clinical practice ability. Although surgical training programs have emphasized a broad-based curriculum intended to train students to pursue a variety of career paths, the most fundamental purpose is to improve students’ ability to solve clinical problems. The clinical practice ability involves many aspects, including history taking, physical examination, communication, diagnosis and differential diagnosis, laboratory examination, and treatment planning [20]. These abilities are necessary to help them engage in competent clinical work as interns as well as improve patient outcomes [57]. However, achieving these abilities requires continuous effort over a long time, and short-term training can only teach them the principles of the work process. The traditional Halsted teaching method of “see one, do one, teach one” in surgical training has lost favor due to patient safety concerns among other reasons [58]. Other adaptations include “See one, simulate many, do one, teach one” which is based on consistent data suggesting that simulation improves resident performance measures of simulation-based tasks [25]. In turn, medical simulation programs such as the use of fresh frozen cadavers, and virtual reality have filled the gap to ensure should continue being explored to improve quantitative performance measures in the operating theatre or patient outcomes. Also, in line with Vygotsky’s concept of “zone of proximal development” [28], the participating students were challenged to assume internist duties. This pushes them over the zone of proximal development frontier where they cannot perform without expert guidance (constructive friction). The physicians’ or internist’s tasks are “zone of proximal development” activities for senior medical students [27]. Under the Vygotskyan concept, destructive friction happens when the gap between the student’s comfort zone and the task is so large that they cannot complete the activity (even with support). Destructive friction also happens when the gap is too narrow (instruction is redundant and might even hamper completion of the activity) [59]. In the present study, this theoretical framework provided guidance on how the students who did not feel confident performing internship level tasks be guided by experts to exit their comfort zones into mastery of new clinical and surgical skills. As posited by Sadideen and colleagues, each student’s zone of proximal development varies thereby demanding dissimilar levels of peer-support and surgical expert-prompting, until eventually the skill can be mastered [60]. Using such an approach enabled the present group of study participants to explore their own development while still in their comfort zone, before continuing to the level of autonomy in performing clinical functions such as patient assessment, appendectomy or suturing.

In the present clinical skills training program, the students were allowed to participate as patients and the rest of the operation theatre team. They could feel the patient’s condition, and the role-play strengthened students’ disease experience. The present results also showed that the students’ core competencies had significantly improved, mainly reflected in the improvement of medico-anatomical knowledge, surgical skills, and the ability to communicate with patients. These observations demonstrated that they were now familiar with the medical processes after undergoing surgical boot camp training. In other words, the present surgical boot camp provided a platform to integrate preclinical anatomy knowledge into medical-surgical practice as well as improved confidence among senior medical students. These benefits of “Boot Camps” on improving clinical skills, knowledge, and confidence were also noted in a recent meta-analysis [31, 42]. Future studies must include control groups, limit heterogeneity in the design as well as evaluation of boot camps studies, the range of outcome measures, and the dearth of long-term follow-up data.

One of the most important aspects of surgical medical education is the training of practical surgical skills. In the present study and teaching module, two methods of surgical skills training were integrated: anatomical dissections and simulated surgery on fresh frozen cadavers. The design of the present model was motivated by the Vygotsky’s concept of “zone of proximal development” [28]. Under this concept, medical students learned surgical skills through the stages that involved initial help and modeling by the surgeons and anatomy teachers. This was gradually replaced with own task performance with dwindling outside help and feedback. Unlike other training programs that used virtual simulation [61, 62], the current students used fresh frozen cadavers to perform specific operations according to the teaching plan. The fresh frozen cadavers greatly preserve the authenticity of the anatomical structures and closely mimic the real clinical experience felt in an operation [63]. In particular, anatomical dissections were added to the curriculum to improve students’ anatomical knowledge for the separation and identification of structures in an operation [38]. Their operative performance showed that the surgical boot camp not only trains them in the practical surgical skills, but also strengthened the rational use of surgical instruments as judged from the overall surgical process. For instance, during appendectomy sessions the present students’ overall performance significantly improved as measured by both the mini-CEX and OPRS evaluation scales. These before and after results, although they lack a control group, suggested that students gradually became familiar with the surgical process from determining the incision to separating anatomical structures. They slowly got used to paying more attention to every detail of the operation, such as the location and depth of the incision and the landmark structures. This suggested that they exited the comfort zones into mastery of operations as well as gained autonomy (reduced outside help from peers and teachers) [27]. Surgical skill training such as suture and knotting were included to ensure the smooth overall operation of the simulated operations. In the present training model, the training sessions were staggered over a longer time to allow for integration of different basic and clinical sciences, unlike the typical boot camp courses that are compacted and short-term. Furthermore, the present long-term model brings together anatomical, medical, and surgical sciences, thereby providing a rationale for the adoption of similar models in designing and implementing boot camp-type training in China and abroad.

One limitation to implementing the surgical boot camp training is that the assessment of clinical practice ability could not be performed on real inpatients in the ward. Patient safety issues have seen the proliferation of simulation as adjuncts to healthcare teaching [64]. Due to fear of interfering with normal clinical work, the present study had not arranged for students to enter the wards. Although students could simulate patients’ feelings through role play, this does not replace the experience of the real environment of the hospital ward. Future training needs to improve the students’ real experience through interaction with hospitalized patients. An additional limitation of this study is that open surgery was used to train the students instead of endoscopic surgery. The main reason is that open surgery is among the basic skills or operations the senior students learn and will use during internship rotations in surgery departments. Moreover, endoscopic surgery requires a relatively long time to learn endoscope operation technology which would also raise patient safety issues. The study did not assess the students’ core competencies by tracking their performance in the clinic. So, the data obtained only reflects the clinical practice ability and operation skills during the surgical boot camp, instead of their real performance in the clinic. Most importantly, the present study limitation was a lack of a control group to evaluate the benefits of the surgical boot intervention and is highly recommended in future studies. However, the present medical students’ reported changes in attitude, perception of knowledge, and confidence remain important take-home messages to motivate similar training in other settings and specialties.

## Conclusions

The surgical boot camp curriculum for senior medical students can improve their clinical practice ability, simulated surgical skills, satisfaction, and confidence. Receiving systematic training before entering the clinic could help senior medical students to become competent interns. Under the current medical education in China, short-term training is a good way to integrate basic science and clinical knowledge for medical students as well as facilitate a seamless transition into internship. Therefore, medical schools the world over should continue to seek ways to bridge the gaps between pre-clinical, clinical, and internship training.

## Supplementary Information

**Additional file 1.**

**Additional file 2.**

## Data Availability

Data used in this study are available on reasonable request from the corresponding author.
