# Supplementary material for: The use of a surgical boot camp combining anatomical education and surgical simulation for internship preparedness among senior medical students
Source: BMC Med Educ. 2022 Jun 15;22:459. doi: 10.1186/s12909-022-03536-y (PMC9202198; doi:10.1186/s12909-022-03536-y)
Supplement: Supplementary file 1 — Additional file 1. [file 12909_2022_3536_MOESM1_ESM.zip › APPENDIX A (Curriculum Topics).docx]

**APPENDIX A**

Curriculum Topics

| Session | Session Time (h) |
| --- | --- |
| *Session 1: Lectures for urgent patient management* |  |
| How to be a good intern | 1 |
| Intern's responsibilities | 1 |
| How to communicate with patients | 1 |
| Pre and postoperative management | 2 |
| Anatomy and surgery | 4 |
| Infection and pain management | 1 |
| Blood transfusion and its principles | 1 |
| Management of surgical complications | 1 |
| Anesthesia and surgery | 1 |
| History and future of surgery | 1 |
| *Session 2: Clinical practice simulation* |  |
| Physical examination | 4 |
| Abdominal puncture and central venous catheterization | 2 |
| Radiological examination | 1 |
| Laboratory examination | 1 |
| *Session 3: Anatomical dissection* |  |
| Abdominal anatomy | 8 |
| Seminar on clinical applied anatomy of abdomen | 2 |
| *Session 4: Surgical simulation* |  |
| Appendectomy | 2 |
| Cholecystectomy | 2 |
| Splenectomy | 2 |
| Intestinal anastomosis | 2 |
| Inguinal hernia repair | 4 |
| Subtotal gastrectomy | 2 |

44
