# Supplementary material for: The use of a surgical boot camp combining anatomical education and surgical simulation for internship preparedness among senior medical students
Source: BMC Med Educ. 2022 Jun 15;22:459. doi: 10.1186/s12909-022-03536-y (PMC9202198; doi:10.1186/s12909-022-03536-y)
Supplement: Supplementary file 1 — Additional file 1. [file 12909_2022_3536_MOESM1_ESM.zip › APPENDIX A to C.docx]

**APPENDIX A**

Curriculum Topics

| Session | Session Time (h) |
| --- | --- |
| *Session 1: Lectures for urgent patient management* |  |
| How to be a good intern | 1 |
| Intern's responsibilities | 1 |
| How to communicate with patients | 1 |
| Pre and postoperative management | 2 |
| Anatomy and surgery | 2 |
| Infection and pain management | 1 |
| Blood transfusion and its principles | 1 |
| Management of surgical complications | 1 |
| Anesthesia and surgery | 1 |
| History and future of surgery | 1 |
| *Session 2: Clinical practice simulation* |  |
| Physical examination | 4 |
| Abdominal puncture and central venous catheterization | 2 |
| Radiological examination | 1 |
| Laboratory examination | 1 |
| *Session 3: Anatomical dissection* |  |
| Abdominal anatomy | 8 |
| Seminar on clinical applied anatomy of abdomen | 2 |
| *Session 4: Surgical simulation* |  |
| Appendectomy | 2 |
| Cholecystectomy | 2 |
| Splenectomy | 2 |
| Intestinal anastomosis | 2 |
| Inguinal hernia repair | 4 |
| Subtotal gastrectomy | 2 |

44

**APPENDIX B**

**Boot Camp Survey Responses**

Name: ___________________

Did you attend a Surgery Boot Camp at your medical school? Y/N

Rate how comfortable are you in the following settings or in managing the following conditions on a scale 1-5 according to the following scale:

•1= strongly disagree.

•2= disagree.

•3= neither disagree nor agree.

•4= agree.

•5= strongly agree.

| Scale |
| --- |
| 1 2 3 4 5 |
| *Facilities of curriculum* |
| Do you have enough time to prepare the curriculum |
| Does the teacher provide enough case information |
| Does the curriculum provide the convenient equipment |
| *Content of the curriculum* |
| Is the curriculum module suitable for you |
| Does surgical boot camp give you valuable clinical practice experience |
| *Clinical lectures* |
| Do the lectures cover the main aspects of clinical practice |
| Are the lectures suitable for you |
| *Clinical practice simulation* |
| Is the content of the clinical practice module rich to meet your needs |
| Does the role-playing train clinical practice skills |
| Do you think the curriculum needs to be arranged to enter the ward to experience the real scene |
| *Anatomical dissections* |
| Is the cadaver dissection training suitable for your needs |
| Does the cadaver dissection session meet your need for anatomical knowledge |
| Does the lecture on clinical applied anatomy cover the main content of the abdomen |
| *Operation simulation* |
| Is the surgical technical training being suitable for your needs |
| Does the operation simulation meet your needs for training surgery |
| Are you satisfied with open surgery |
| Is it necessary to add endoscopic surgery simulation to the curriculum |
| *Overall satisfaction* |
| Are you satisfied with the whole curriculum |

**Mini-Clinical Evaluation Exercise (CEX)**

Evaluator: Date:

Resident: Grade:

Patient Problem：

Setting：Ambulatory□ In-patient□ ED□ Other□

Patient：Age: Sex: New: Follow-up:

Complex: Low□ Moderate□ High□

Focus: Data Gathering□ Diagnosis□ Therapy□ Counseling□

**1. Medical Interviewing Skills** **(Not observed**□)

| 1 | 2 | 3 | 4 | 5 | 6 | 7 | 8 | 9 |
| --- | --- | --- | --- | --- | --- | --- | --- | --- |
| Unsatisfactory | | | Satisfactory | | | Superior | | |

**2. Physical Examination Skills (Not observed**□)

| 1 | 2 | 3 | 4 | 5 | 6 | 7 | 8 | 9 |
| --- | --- | --- | --- | --- | --- | --- | --- | --- |
| Unsatisfactory | | | Satisfactory | | | Superior | | |

**3. Humanistic Qualities/Professionalism(Not observed**□)

| 1 | 2 | 3 | 4 | 5 | 6 | 7 | 8 | 9 |
| --- | --- | --- | --- | --- | --- | --- | --- | --- |
| Unsatisfactory | | | Satisfactory | | | Superior | | |

**4. Clinical Judgment(Not observed**□)

| 1 | 2 | 3 | 4 | 5 | 6 | 7 | 8 | 9 |
| --- | --- | --- | --- | --- | --- | --- | --- | --- |
| Unsatisfactory | | | Satisfactory | | | Superior | | |

**5. Counseling Skills(Not observed**□)

| 1 | 2 | 3 | 4 | 5 | 6 | 7 | 8 | 9 |
| --- | --- | --- | --- | --- | --- | --- | --- | --- |
| Unsatisfactory | | | Satisfactory | | | Superior | | |

**6. Organization/Efficiency(Not observed**□)

| 1 | 2 | 3 | 4 | 5 | 6 | 7 | 8 | 9 |
| --- | --- | --- | --- | --- | --- | --- | --- | --- |
| Unsatisfactory | | | Satisfactory | | | Superior | | |

**7. Overall Clinical Competence(Not observed**□)

| 1 | 2 | 3 | 4 | 5 | 6 | 7 | 8 | 9 |
| --- | --- | --- | --- | --- | --- | --- | --- | --- |
| Unsatisfactory | | | Satisfactory | | | Superior | | |

Mini-CEX Time: Oberserving Mins; Providing Feedback Mins

Evaluator Satisfaction with Mini-CEX：Low 1 2 3 4 5 6 7 8 9 High

Resident Satisfaction with Mini-CEX：Low 1 2 3 4 5 6 7 8 9 High

Comments：

Resident Signature: Ev
