# Supplementary material for: The use of a surgical boot camp combining anatomical education and surgical simulation for internship preparedness among senior medical students
Source: BMC Med Educ. 2022 Jun 15;22:459. doi: 10.1186/s12909-022-03536-y (PMC9202198; doi:10.1186/s12909-022-03536-y)
Supplement: Supplementary file 1 — Additional file 1. [file 12909_2022_3536_MOESM1_ESM.zip › APPENDIX C (Mini-CEX).docx]

**Mini-Clinical Evaluation Exercise (CEX)**

Evaluator: Date:

Resident: Grade:

Patient Problem：

Setting：Ambulatory□ In-patient□ ED□ Other□

Patient：Age: Sex: New: Follow-up:

Complex: Low□ Moderate□ High□

Focus: Data Gathering□ Diagnosis□ Therapy□ Counseling□

**1. Medical Interviewing Skills** **(Not observed**□)

| 1 | 2 | 3 | 4 | 5 | 6 | 7 | 8 | 9 |
| --- | --- | --- | --- | --- | --- | --- | --- | --- |
| Unsatisfactory | | | Satisfactory | | | Superior | | |

**2. Physical Examination Skills (Not observed**□)

| 1 | 2 | 3 | 4 | 5 | 6 | 7 | 8 | 9 |
| --- | --- | --- | --- | --- | --- | --- | --- | --- |
| Unsatisfactory | | | Satisfactory | | | Superior | | |

**3. Humanistic Qualities/Professionalism(Not observed**□)

| 1 | 2 | 3 | 4 | 5 | 6 | 7 | 8 | 9 |
| --- | --- | --- | --- | --- | --- | --- | --- | --- |
| Unsatisfactory | | | Satisfactory | | | Superior | | |

**4. Clinical Judgment(Not observed**□)

| 1 | 2 | 3 | 4 | 5 | 6 | 7 | 8 | 9 |
| --- | --- | --- | --- | --- | --- | --- | --- | --- |
| Unsatisfactory | | | Satisfactory | | | Superior | | |

**5. Counseling Skills(Not observed**□)

| 1 | 2 | 3 | 4 | 5 | 6 | 7 | 8 | 9 |
| --- | --- | --- | --- | --- | --- | --- | --- | --- |
| Unsatisfactory | | | Satisfactory | | | Superior | | |

**6. Organization/Efficiency(Not observed**□)

| 1 | 2 | 3 | 4 | 5 | 6 | 7 | 8 | 9 |
| --- | --- | --- | --- | --- | --- | --- | --- | --- |
| Unsatisfactory | | | Satisfactory | | | Superior | | |

**7. Overall Clinical Competence(Not observed**□)

| 1 | 2 | 3 | 4 | 5 | 6 | 7 | 8 | 9 |
| --- | --- | --- | --- | --- | --- | --- | --- | --- |
| Unsatisfactory | | | Satisfactory | | | Superior | | |

Mini-CEX Time: Oberserving Mins; Providing Feedback Mins

Evaluator Satisfaction with Mini-CEX：Low 1 2 3 4 5 6 7 8 9 High

Resident Satisfaction with Mini-CEX：Low 1 2 3 4 5 6 7 8 9 High

Comments：

Resident Signature: Evaluator Signature:
