# Supplementary material for: The use of a surgical boot camp combining anatomical education and surgical simulation for internship preparedness among senior medical students
Source: BMC Med Educ. 2022 Jun 15;22:459. doi: 10.1186/s12909-022-03536-y (PMC9202198; doi:10.1186/s12909-022-03536-y)
Supplement: Supplementary file 2 — Additional file 2. [file 12909_2022_3536_MOESM2_ESM.docx]

**APPENDIX D**

**Boot Camp Survey Responses**

Name: ___________________

Did you attend a Surgery Boot Camp at your medical school? Y/N

Rate how comfortable are you in the following settings or in managing the following conditions on a scale 1-5 according to the following scale:

•1= strongly disagree.

•2= disagree.

•3= neither disagree nor agree.

•4= agree.

•5= strongly agree.

| Scale |
| --- |
| 1 2 3 4 5 |
| *Facilities of curriculum* |
| Do you have enough time to prepare the curriculum |
| Does the teacher provide enough case information |
| Does the curriculum provide the convenient equipment |
| *Content of the curriculum* |
| Is the curriculum module suitable for you |
| Does surgical boot camp give you valuable clinical practice experience |
| *Clinical lectures* |
| Do the lectures cover the main aspects of clinical practice |
| Are the lectures suitable for you |
| *Clinical practice simulation* |
| Is the content of the clinical practice module rich to meet your needs |
| Does the role-playing train clinical practice skills |
| Do you think the curriculum needs to be arranged to enter the ward to experience the real scene |
| *Anatomical dissections* |
| Is the cadaver dissection training suitable for your needs |
| Does the cadaver dissection session meet your need for anatomical knowledge |
| Does the lecture on clinical applied anatomy cover the main content of the abdomen |
| *Operation simulation* |
| Is the surgical technical training being suitable for your needs |
| Does the operation simulation meet your needs for training surgery |
| Are you satisfied with open surgery |
| Is it necessary to add endoscopic surgery simulation to the curriculum |
| *Overall satisfaction* |
| Are you satisfied with the whole curriculum |
